# Circulating and disseminated tumor cells: harbingers or initiators of metastasis?

**DOI:** 10.1002/1878-0261.12022

**Published:** 2017-01-09

**Authors:** Arko Dasgupta, Andrea R. Lim, Cyrus M. Ghajar

**Affiliations:** ^1^ Public Health Sciences Division/Translational Research Program and Human Biology Division Fred Hutchinson Cancer Research Center Seattle WA USA; ^2^ Program in Molecular and Cellular Biology University of Washington Seattle WA USA

**Keywords:** chemoresistance, circulating tumor cells, disseminated tumor cells, dormancy, metastatic microenvironment, perivascular niche

## Abstract

Tumor cells leave the primary tumor and enter the circulation. Once there, they are called circulating tumor cells (CTCs). A fraction of CTCs are capable of entering distant sites and persisting as disseminated tumor cells (DTCs). An even smaller fraction of DTCs are capable of progressing toward metastases. It is known that the DTC microenvironment plays an important role in sustaining their survival, regulating their growth, and conferring resistance to therapy. But we still have much to learn about the nature of these rare cell populations to predict which will progress and what exactly should cause concern for future relapse. Although recent technological advances in our ability to detect and molecularly and functionally characterize CTCs and DTCs promise to unravel this ambiguity, the timing of dissemination and the precise source of CTCs and DTCs profiled will impact the conclusions that can be made from these endeavors. In this review, we discuss the biology of CTCs and DTCs; the technologies to detect, isolate, and profile these cells; and the exceptions we must apply to our understanding of what role these cells play in the metastatic process. We conclude that a greater effort to understand the unique biology of these cells in context will positively impact our ability to use these cells to predict outcome, monitor treatment efficacy, and reveal therapeutically relevant targets to deplete these populations and ultimately prevent metastasis.

**Abbreviations**
AML: acute myeloid leukemia
BMP: bone morphogenic protein
CDXs: CTC‐derived explants
CGH: comparative genome hybridization
CTCs: circulating tumor cells
DCIS: ductal carcinoma *in situ*
DTCs: disseminated tumor cells
ECM: extracellular matrix
EGFR: epithelial growth factor receptor
EMT: epithelial‐to‐mesenchymal transition
EpCAM: epithelial cell adhesion molecule
ERBB2: erb‐B2 receptor tyrosine kinase 2
ER: estrogen receptor
G‐CSF: granulocyte colony‐stimulating factor
HER2: human epidermal growth factor receptor 2
HSCs: hematopoietic stem cells
ICC: immunocytochemistry
IHC: immunohistochemistry
ISH: *in situ* hybridization
LIF: leukemia inhibitory factor
MET: mesenchymal‐to‐epithelial transition
MHCI: major histocompatibility complex I
MICs: metastasis‐initiator cells
MMPs: matrix metalloproteinases
NKG2D: natural killer group 2D
NSCLC: non‐small‐cell lung cancer
PCR: polymerase chain reaction
PVN: perivascular niche
RNA‐ISH: RNA *in situ* hybridization
RNA‐seq: RNA sequencing
RT‐PCR: real‐time polymerase chain reaction
SCLC: small cell lung cancer
TGF‐β2: transforming growth factor‐β2
TGF‐β: transforming growth factor‐β
TSP‐1: thrombospondin‐1

## 1 Introduction

Perhaps the most important discovery in the last 15 years of basic metastasis research is the finding that tumor cells disseminate at the so‐called *in situ* stage (Braun *et al*., 2005; Gruber *et al*., 2016; Sänger *et al*., 2011), well before detection of the primary tumor. In retrospect, this should not have been so surprising. Five to ten percent of all cases of metastatic cancer are cancer of unknown primary (Abbruzzese *et al*., 1994; Klein, 2009; Massard *et al*., 2011; van de Wouw *et al*., 2002), where the metastatic lesion is detected before the primary tumor. The implications of early dissemination – that disseminated tumor cells (DTCs) a) lack many of the genomic abnormalities that characterize the primary tumor, and b) evolve in parallel with the primary tumor – have great relevance now that we are at the dawn of personalized medicine.

The question is how to instigate a paradigm shift in metastasis research so that personalized medicine is applied to DTCs and their circulating counterparts, CTCs, rather than solely to the primary tumor. How do we shift the focus from treating metastasis to preventing metastasis when we currently do not have any drugs that specifically target DTCs or CTCs? What approaches are necessary to identify actionable targets that could lead to CTC and/or DTC depletion? This is essentially the only way to test the logical assumption that successful depletion of these cell populations would result in prolonged metastasis‐free survival. And lastly, once we successfully develop these approaches, we must understand heterogeneity within and among patients so that we can determine which patients would benefit from such therapies.

Addressing these challenges not only requires bold thinking, it necessitates the development of technologies and models to study rare cell populations in context. In this review, we shine a spotlight on these harbingers of metastasis with a focus on early stages of the disease. We highlight the biology that implicates CTCs and DTCs in metastasis; the technologies and models developed to detect, profile, and study these cells; the clinical implications of current findings; and what is left to uncover.

## 2 Biology of CTCs

The study of CTC biology is simplified by the fact that CTCs can be isolated from blood using a standard blood draw. Several new high‐throughput technologies have been developed to isolate these cells from the blood and study them at the single‐cell level. CTCs are considered to be en route to or from disseminated sites and reflective of metastasis, and several studies have shown their diagnostic and prognostic significance.

Attempts to further develop CTC isolation, detection, and analysis techniques as clinical platforms to aid disease management are underway. The information gained from CTCs isolated over the course of disease or treatment can provide a more dynamic, therapeutically relevant, and patient‐centric means to treat cancer. We will begin with a primer on CTC biology – primarily, the characteristics that facilitate exit from primary tissue and survival through several stressful bottlenecks to reach their site of dissemination.

### 2.1 To leave for the unknown

**Figure 1:**
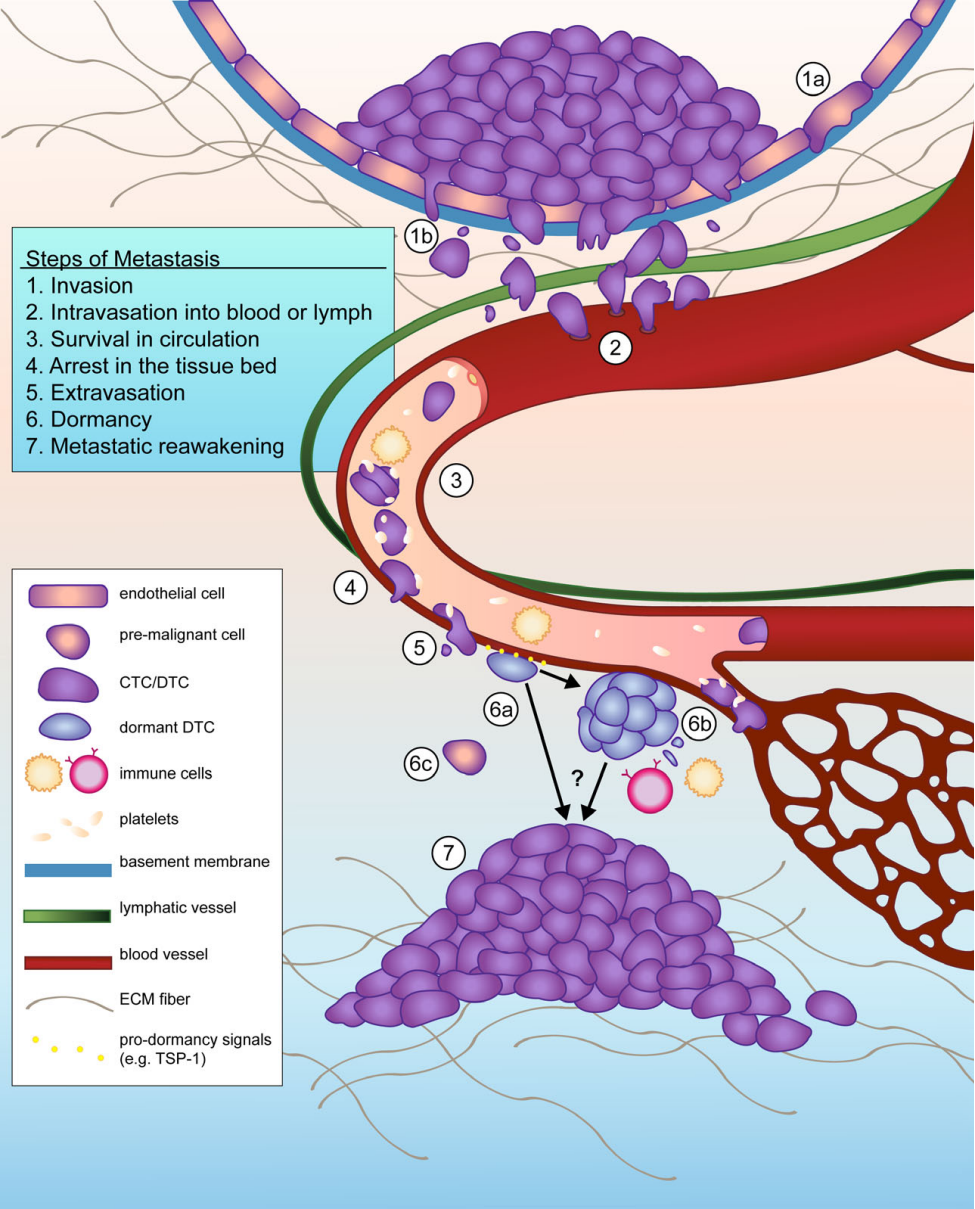
Tumor cell dissemination enables the spread of cancer from its site of origin. Local invasion through the basement membrane of a duct and ECM fibers may occur early (1a) or later (1b) in malignant progression. Upon reaching a local or intratumoral vessel conduit, DTCs intravasate into hematogenous or lymphatic vessels (2). Blood is a hostile environment and CTCs must contend with circulating immune cells, loss of cell–cell junctions, and shear stress to survive (3). Platelets offset these hurdles by providing a protective coat that promotes the formation of tumor microemboli and arrest on the luminal side of capillaries and venules (4). As DTCs extravasate (5), they encounter a foreign microenvironment containing new obstacles to survival. Some DTCs enter dormancy during this stressful transition, a process in which microenvironmental factors such as TSP‐1, BMP‐4, and BMP‐7 have been implicated. Single dormant cells that undergo cell cycle arrest have been observed in the perivascular niche (6a), where they apparently avoid immunodetection. Small DTC clusters and micrometastases may continue to divide, but are prevented from crossing a size threshold due to angiogenic limitations or immunosurveillance (6b). The contribution of early disseminators, which may or may not have ever entered a completely malignant state, is unknown (6c). The mechanisms through which dormant DTCs eventually reawaken are currently being elucidated (7) and likely include tissue‐specific factors, molecules derived from angiogenic neovessels, and other unknown effectors.

Circulating tumor cells must overcome a number of physiological hurdles to disseminate. To enter the circulation, tumor cells must invade from the epithelium or tumor of origin, navigate through their local microenvironment, and traverse the endothelium (intravasation). Once in circulation, CTCs must survive shear and immunological pressures, exit from circulation (extravasation), and successfully incorporate within their new tissue (Fig. 1).

How do tumor cells emigrate from a primary site? One widely prescribed theory is that cells within solid tumors must undergo an epithelial‐to‐mesenchymal transition (EMT) in order to invade (Hanahan and Weinberg, 2011; Labelle *et al*., 2011; Pantel and Speicher, 2015; Yu *et al*., 2013). This phenotypic transition is accompanied by molecular changes that enable epithelial cells to become more invasive, motile, and capable of seeding distant sites (Joosse *et al*., 2015; Yang and Weinberg, 2008). Several of the physical hurdles faced by CTCs can be addressed by the potential of malignant cells (and future CTCs) to undergo EMT. For example, a cell undergoing EMT downregulates E‐cadherin, which enables detachment from neighboring epithelial cells, and upregulates matrix metalloproteinase (MMP) activity, which facilitates navigation through the local extracellular matrix (ECM) and entry to microvasculature (Lamouille *et al*., 2014; Lee *et al*., 2006).

Circulating tumor cells with mesenchymal features predict poor outcome in a number of cancers, indicating that this phenotypic shift provides an advantage in circulation and/or distant sites. In a cohort of 39 patients with metastatic breast cancer, 62% of CTC‐positive patients had CTCs that were positive for at least one of three EMT markers (Akt2, PI3K, and Twist1) multiplexed using RT‐PCR. This comprised the majority of patients that did not respond to standard chemo‐, antibody, or hormonal therapy, as opposed to just 10% in responders (Aktas *et al*., 2009).

The caveat with this study and others like it are the limited number of genes and markers used to define the occurrence of a phenotypic switch that involves much more than a handful of genes (Diepenbruck and Christofori, 2016). Application of gene expression microarrays in a mouse model of breast cancer metastasis showed that the EMT signature detected in CTCs is not limited to one or two transcripts or molecules (LeBleu *et al*., 2014), but instead involves significant changes in expression of several genes, for example, in at least 76 genes in lung cancer (Byers *et al*., 2013). These signatures also evolve over time with treatment. In a study where CTCs were analyzed from an individual patient using RNA *in situ* hybridization (RNA‐ISH), resistance to therapy was accompanied by a shift toward the mesenchymal molecular signature (Yu *et al*., 2013). The dominance of the mesenchymal‐like fraction in this patient's CTCs showed a dramatic decrease when another effective therapy was introduced (Yu *et al*., 2013). Ideally, establishing accurate molecular correlates between CTC phenotype and metastasis‐initiating potential would enable one to monitor treatment efficacy via serial liquid biopsies. Whether EMT constitutes this phenotypic signature on its own remains to be seen.

However, questions have been raised about the obligate requirement of EMT in the generation of cells capable of leaving the primary tumor (Christiansen and Rajasekaran, 2006; Tarin *et al*., 2005). If EMT is necessary for dissemination, then it follows that the reverse, or mesenchymal‐to‐epithelial transition (MET), is necessary for CTCs to ultimately grow within a distant tissue. This would require another overhaul of the transcriptome and proteome in what is already considered an inefficient process (i.e., dissemination). In fact, DTCs from several cancers found in the bone marrow are epithelial in nature (Braun *et al*., 2000; Pantel *et al*., 1996; Schardt *et al*., 2005); perhaps for these early‐stage disseminated cells, this transition never (or only partially) took place. Indeed, under varying experimental conditions, EMT‐associated transcriptional factors have both positive (Yang and Weinberg, 2008; Yang *et al*., 2004) and negative (Fischer *et al*., 2015; Ocaña *et al*., 2012; Tsai *et al*., 2012) effects on metastasis. Cancer cells destined to leave the primary tumor display only a small part of the spectrum of changes associated with EMT in development (Vanharanta and Massagué, 2013; Yang and Weinberg, 2008). Eventually, the specific influence of EMT ‘drivers’ on the release of CTCs from the primary tissue and the ability of mesenchymal‐like cells to seed and survive within distant organs will have to be elucidated.

In addition to individual CTCs, CTC clusters are also found in patient blood. CTC clusters are composed of 2 to more than 50 cells and are relatively rare compared with single cells in circulation. Larger clusters, also called tumor microemboli, have been detected in several cancers using various technologies, and the presence of these microemboli in circulation generally correlates with very poor clinical outcome in lung and breast cancer (Aceto *et al*., 2015; Al‐Mehdi *et al*., 2000; Christiansen and Rajasekaran, 2006). In the case of breast cancer, clusters appear to be oligoclonal in nature and not an artifact of cellular replication in circulation (Aceto *et al*., 2014). This could provide cells within a CTC cluster with a survival advantage over single cells. Indeed, cells in clusters display a stark lack of apoptosis (Hou *et al*., 2012), possibly due to maintaining a basal level of cell junction‐mediated survival signaling that helps them avoid anoikis (Paoli *et al*., 2013). Upon extravasation, autocrine signaling within a cluster may facilitate more rapid adaptation to a new environment in distant organs. CTC clusters may also contain normal cells from the primary site (e.g., stromal cells), which has major implications for the ‘seed‐and‐soil’ hypothesis (Paget, 1889). Bringing its own soil should only enhance the ability of a seed to sprout at distant sites (Aceto *et al*., 2015; Duda *et al*., 2010; Jones *et al*., 2013).

### 2.2 Platelets contribute to the survival of CTCs and clusters

Circulating tumor cells get help from other nontumor entities along the way. For instance, platelets contribute in multiple ways to CTC persistence in blood. Experiments in mouse models with no circulating platelets (e.g., NF‐E2−/− mice), nonfunctional platelets (e.g., Par4−/− mice), or a reduced number of platelets following treatment with neuraminidase have shown that lack of appropriate platelet function hampers metastasis (Camerer *et al*., 2004; Gasic *et al*., 1968; Pearlstein *et al*., 1980). The prometastatic effects of platelets are both physical and molecular. From a mechanical perspective, platelets rapidly coat CTCs in the bloodstream, shielding them from violent shear forces (McCarty *et al*., 2000; Nieswandt *et al*., 1999) (Fig. 1, Step 3). Tumor cells bind platelet adhesive proteins fibronectin and von Willebrand factor via integrins, promoting crosslinking and coagulation (Nierodzik *et al*., 1991). Tumor cell‐induced platelet aggregates result in tumor emboli, which have a greater chance of entrapment in small vessels, allowing more time for extravasation to occur. Further, platelets promote adhesion to the luminal side of the endothelia via surface molecules such as selectins (Läubli and Borsig, 2010).

Platelets also provide a defense against the immune system. For instance, platelet‐derived TGF‐β inactivates natural killer cells via downregulation of NKG2D (Kopp *et al*., 2009). Immune attenuation also occurs through transfer of MHC I complexes from granulated platelets to CTCs, endowing the latter with a ‘self’ identity that discourages natural killer cell‐mediated cytolytic attack (Placke *et al*., 2012).

Despite all of this help, CTCs persist in circulation for only 1–2.4 h according to *in vivo* experimental modeling and data from a small cohort of five patients (Meng *et al*., 2004). Most CTCs die in circulation as a result of shear stress and/or anoikis (Labelle and Hynes, 2012; Vanharanta and Massagué, 2013). Paradoxically, the half‐life of CTC clusters in circulation has been reported to be shorter than that of single CTCs, but this may be because large clusters get ‘cleared’ from the bloodstream or get stuck in capillaries more often than single CTCs (Aceto *et al*., 2014). Theoretically, survival signaling due to cell–cell junction‐derived signaling, reduction in shear stress experienced by individual components of the cluster, and longer dwell time in tiny capillaries should promote viability of CTC clusters versus single CTCs.

## 3 Isolation and characterization of CTCs

### 3.1 CTC isolation

Although highly aggressive tumors shed thousands of cancer cells into circulation each day (Allard *et al*., 2004; Nagrath *et al*., 2007), CTCs comprise a fairly rare population within blood. Technologies developed over the past two decades to detect CTCs are generally based on properties that either define CTCs (positive selection) or are uncharacteristic of CTCs (negative selection), as reviewed thoroughly elsewhere (Aceto *et al*., 2015; Joosse *et al*., 2015; Krebs *et al*., 2014; Millner *et al*., 2013). CTCs originating from epithelial tumors are thought by many to exclusively express epithelial cell adhesion molecule (EpCAM; a faulty assumption that is discussed below). EpCAM selection underlies the basic strategy of the CellSearch system, which has been approved by the US FDA for use in breast, colorectal, and prostate cancer. More recent technologies such as the micropost‐based CTC chip and the herringbone (HB)‐based CTC chip allow increased sensitivity due to the incorporation of additional epithelial tumor antigens such as HER2 (human epidermal growth factor receptor 2) and EGFR (epithelial growth factor receptor) (Nagrath *et al*., 2007; Stott *et al*., 2010; Yu *et al*., 2013).

Given the relatively rare nature of CTCs and CTC clusters, GILUPI Nanomedizin developed a technology that enables sampling of CTCs *in vivo* by inserting an EpCAM‐coated detection wire into the antecubital vein for 30 min. This allows the probing of a large volume of blood to isolate CTCs and CTC clusters (Saucedo‐Zeni *et al*., 2012). Essentially the opposite approach – diagnostic leukapheresis – has been used to sample much larger volumes of blood to overcome issues with detection that occur due to the rare nature of the CTCs (Fischer *et al*., 2013).

Regardless of the approach, it is important to remember that tumor classifications represent population averages; therefore, one cannot expect all CTCs to express a primary tumor marker over a threshold level. Indeed, a study using breast cancer cell lines showed that approximately 20% of cell lines expressed EpCAM at low levels. These cells instead showed a basal‐like phenotype with lower overall expression of epithelial markers and increased expression of mesenchymal markers (Punnoose *et al*., 2010). This datum suggests that mesenchymal‐like cells may be missed or undersampled using only EpCAM to select for CTCs (Punnoose *et al*., 2010). This is important for prognostic purposes because EpCAM^−^ circulating breast tumor cells may actually constitute a Her2^+^/EGFR^+^/HPSE^+^/Notch1^+^ population with enhanced propensity to colonize brain (Zhang *et al*., 2013). On the other hand, EpCAM‐based selection also falsely identifies normal cells as tumor cells. Erythroid progenitor cells in the bone marrow express EpCAM transiently (Lammers *et al*., 2002), and this has been taken into consideration in the definition of a dormancy signature of EpCAM^+^ disseminated prostate tumor cells in bone marrow (Chéry *et al*., 2014).

Antigen‐independent methods have also been developed to isolate and study CTCs. The CTC‐iCHIP offers an improved technology by separating out blood components through hydrodynamic separation followed by immune depletion to deliver purified CTCs that have not been tagged and manipulated (Ozkumur *et al*., 2013). Furthermore, some technologies work optimally with CTC clusters when compared with the previously mentioned technologies that are limited to single cells or two to four cell clusters. Technologies such as the ‘cluster chip’ have been developed to take into account the need to isolate structurally sound and viable CTC clusters for downstream studies (Sarioglu *et al*., 2015).

### 3.2 Molecular characterization of CTCs

Isolation of viable CTCs enables their molecular and functional characterization. Chromosomal rearrangements or changes in CTC gene copy number can be studied using techniques such as interphase fluorescence *in situ* hybridization (ISH) and array‐CGH (comparative genome hybridization). Additionally, next‐generation DNA sequencing allows the study of the genome‐wide mutation spectrum in CTCs. Using a combination of array‐CGH and next‐generation sequencing, several cancer‐associated copy number changes shared with the primary tumor were described in 37 intact CTCs isolated from six patients with colorectal cancer (Heitzer *et al*., 2013). Comparison of the mutation spectrum among tumors, CTCs, and metastases using massive parallel sequencing in two of these patients revealed not only the expected ‘driver mutations’, but also CTC‐specific mutations initially. However, it was subsequently confirmed that these ‘CTC‐specific’ mutations were in fact present also in subclonal levels in the primary tumor and in metastases (Heitzer *et al*., 2013). This study serves as an example of the power of sequencing technologies available to researchers to study CTC biology in the context of systemic disease. Coupled with its ever‐reducing cost, sequencing technologies could eventually transform patient‐specific treatment over the course of systemic disease.

Whereas traditional techniques such as RNA *in situ* hybridization with limited pooling of targets, multiplexed quantitative PCR, and gene expression microarrays have been utilized to study CTCs (Payne *et al*., 2012; Smirnov *et al*., 2005; Xi *et al*., 2007; Yu *et al*., 2013), single‐cell expression profiling approaches are coming to the fore given the relative rarity of CTCs and the limited amount of genetic material one can isolate from them (Sandberg, 2013). Powerful techniques such as single nucleus (cell) sequencing and whole‐exome sequencing have been employed to study clonal composition among primary breast cancer cells and prostate cancer‐derived CTCs (Lohr *et al*., 2014; Wang *et al*., 2014). These studies shed light on the heterogeneity of CTCs with respect to time and response to therapy. For example, the application of RNA‐Seq technology to single prostate CTCs (77 intact CTCs from 13 patients) showed considerable heterogeneity between CTCs from a single patient, and identified noncanonical WNT signaling as a mechanism of resistance to therapies targeting androgen receptor (Miyamoto *et al*., 2015).

It remains to be seen if such assays will become predictive, specific, and inexpensive enough to be used routinely. Although it would require a technological leap, one can envision single‐cell proteomic approaches being applied to CTCs eventually in order to measure antigen diversity in metastatic disease or to identify targets for immunotherapy in the context of minimal residual disease.

### 3.3 Functional characterization of CTCs

Functional characterization of CTCs is technically challenging due to their rarity. Thus, developing standardized techniques to culture CTCs isolated from patients with different types of cancer is critical, keeping in mind that the changes CTCs undergo after isolation and passaging (particularly if immortalized) cast a shadow on subsequent results. Cell lines and organoid cultures have been established from CTCs, but with very low efficiency and success. Such endeavors have come to fruition only for a handful of cancers (e.g., Cayrefourcq *et al*., 2015).

Circulating tumor cell functionality has been tested in culture and in mice, such as an assay where CTCs are plated on fluorescently labeled ECM in order to measure invasiveness (Friedlander *et al*., 2014) or xenotransplantation of CTCs into immunodeficient mice. The tumors that grow out in these mice, called CTC‐derived explants (CDXs), could serve as vital material for the choice or design of therapeutic regimens. Xenotransplantation experiments using luminal breast cancer patient‐derived CTCs identified an EPCAM^low^MET^high^CD47^high^CD44^high^ population proposed to be metastasis‐initiator cells (MICs) (Baccelli *et al*., 2013). Utilizing a CDX model of small cell lung cancer (SCLC), one group identified a CTC profile that predicted response to platinum‐based drugs and etoposides (Hodgkinson *et al*., 2014).

It should be noted, however, that *in vivo* strategies used to study CTCs lack uniformity in one important aspect – the site of injection. In the breast cancer and SCLC studies mentioned previously, CTCs were injected into the femoral medullary cavity and flanks of immunocompromised mice, respectively (Baccelli *et al*., 2013; Hodgkinson *et al*., 2014). Injection sites for CTCs are chosen often for technical reasons governed by the number of CTCs at hand, the number of assays to be performed, and in‐house expertise. However, there needs to be careful consideration about artifacts that are introduced by the choice of these injection sites, which clearly comprise very different microenvironments. For example, injecting patient‐derived CTCs into solid tissue (e.g., the flanks of mice) could provide a survival advantage by eliminating the need to a) survive in circulation, b) extravasate as an individual cell, and c) survive and outgrow from an individual cell in the absence of potentially stimulating factors from other tumor cells.

## 4 Clinical relevance of CTCs: does timing of detection matter?

A number of studies have sought to validate the degree to which CTCs mirror their tumor of origin and the metastases they initiate. The presence of CTCs in blood negatively correlates with progression‐free and overall survival at early stages of lung cancer, gastric cancer, melanoma, and pancreatic cancer (Ilie *et al*., 2014; Mimori *et al*., 2008; Reid *et al*., 2013; Rhim *et al*., 2014). Accumulating evidence suggests that CTCs are detectable at very early stages in multiple types of cancer, yet the discrepancy between the number of metastatic lesions and the number of detectable CTCs suggests that not all CTCs are created equal with regard to colony‐forming potential.

The metastasis‐initiating capacity of early CTCs at the neoplastic or hyperplastic stage of primary tumor development is not well characterized (Baccelli *et al*., 2013; Tsai *et al*., 2016); however, given that 5–10% of metastases come from primary tumors of unknown origin, early CTCs are clearly capable of colonizing distant sites and thus are important to the timeline of metastatic progression (van de Wouw *et al*., 2002). Indeed, a CTC screen conducted on healthy individuals who were, nonetheless, positive for an independent risk factor of non‐small‐cell lung cancer (NSCLC) led to the detection of CTCs in a subset of this cohort (Ilie *et al*., 2014). The CTC‐positive individuals all developed lung nodules within 4 years of CTC detection, prompting immediate surgical intervention.

Whether the relevance of CTCs depends on cancer type remains to be confirmed, especially in light of accumulating data that certain cancer types tend to disseminate early (discussed in greater detail in Section 3.1) (Klein, 2009). Further, early CTC detection may be confounded by normal or benign circulating epithelial cells, as is the case with inflammatory colorectal diseases (Pantel *et al*., 2012). The development of panels including epithelial, mesenchymal, and tissue of origin markers may help filter out normal circulating epithelial cells and avoid false positives.

Recent work also linked CTC number with primary tumor size, invasiveness (lymph node status), presence of metastasis, and clinical outcome (Ma *et al*., 2012; Mocellin *et al*., 2006; Rahbari *et al*., 2010; Wang *et al*., 2015; Zhang *et al*., 2012). Detecting as few as two to five CTCs per 7.5–10 mL blood predicts shorter progression‐free and overall survival across cancer types and stages (Cohen *et al*., 2008; Cristofanilli *et al*., 2004; Hiraiwa *et al*., 2008; Karhade *et al*., 2014; Rack *et al*., 2014; Thalgott *et al*., 2013). CTCs may also be useful predictors of metastatic burden. Metastatic patients are more likely to be CTC‐positive than nonmetastatic patients in the case of prostate and GI cancer (Hiraiwa *et al*., 2008; Thalgott *et al*., 2013), whereas higher CTC count predicts liver metastases from pancreatic adenocarcinoma (Tien *et al*., 2016). Therefore, defining CTC thresholds would be useful to predict patients at risk for recurrence. Developing means to faithfully monitor disease progression, recurrence, and mechanisms of resistance using CTCs will direct us toward successful clinical avenues for both treatment and prevention.

## 5 Lost in transition: CTCs and DTCs in the context of early dissemination

If the technologies to detect and characterize CTCs are to become routine in the prevention of disease, then the timing of their application needs to be carefully optimized. Mounting evidence for the parallel progression of cancer (Klein, 2009) argues that knowing the species (primary tumor or DTC) contributing to the CTC pool is important to accurately predict progression and/or response to therapy.

Tumor cells leave the primary site well before the primary tumor is clinically established. In fact, pancreatic cancer CTCs with mesenchymal and stem cell‐like phenotypes can be detected in the circulation of mouse of models even before detection of the primary tumor (Rhim *et al*., 2012). Additionally, some of these early DTCs seed the liver at this early stage, suggesting that early‐stage DTCs have the ability to not only leave the primary site but also successfully seed a secondary distant site (and perhaps even re‐enter the circulation). Blood‐borne dissemination is also an early event in patients with breast cancer, as DTCs have been detected in patients with preinvasive ductal carcinoma *in situ* (DCIS) (Gruber *et al*., 2016; Sänger *et al*., 2011), a phenomenon that has been recapitulated in detail in mouse models (Hüsemann *et al*., 2008).

What does early dissemination mean from the perspective of utilizing CTCs as a diagnostic and prognostic tool? There is a distinct possibility that chronologically disparate CTCs differ at the molecular level. Indeed, CTCs that disseminate early and form micrometastatic lesions undetectable by standard clinical imaging technologies may actually be comprised of a greater number of MICs than those derived later from frank lesions. But the CTC compartment may be dominated by contributions from the primary tumor, which contains several orders of magnitude more cells, at the time of diagnosis, essentially masking the metastasis‐initiating population that may be more critical to monitor. It should be noted that currently, there is no concrete evidence that CTCs detected later in disease are more (or less) capable of disseminating and forming metastases than early CTCs. Testing such a hypothesis is technically challenging due to the small number of CTCs that can be isolated from patients and animal models; however, investigating the proportion of early CTCs that are dissemination‐ and metastasis‐competent compared with late CTCs warrants exploration given that early DTCs have been shown to form metastases (Hüsemann *et al*., 2008).

A final, related caveat is that substantial heterogeneity exists in the techniques used to isolate and characterize CTCs. For instance, the location of sample collection varies widely from study to study. CTC count (and potentially source) varies with the blood compartment from which it is drawn, that is, right versus left side of the heart (Rhim *et al*., 2012) or mesenteric versus venous blood (Rahbari *et al*., 2012). These observational differences may underlie a natural enrichment of CTCs in somatic distribution based on circulation patterns.

## 6 Biology of DTCs

The vast majority of cancer‐associated mortalities are not due to the primary tumor but because of the presence of tumor emboli or metastatic lesions at distant sites, which often cause organ failure. CTCs may seed these distant sites, but even if they survive, the dynamics of outgrowth vary considerably between cells, cancer types, and individual patients. Indeed, in melanoma, breast, and prostate cancer, DTCs may enter into a quiescent state, only to ‘awaken’ after years or even decades and form deadly metastases (Chambers *et al*., 2002).

Long considered a ‘black box’ in the metastatic cascade, progress is being made to understand DTC biology. Evidence is accumulating that the DTC microenvironment plays a significant role in determining their signature properties: prolonged survival, reversible growth arrest, and therapeutic resistance (Ghajar, 2015).

### 6.1 Dormancy: the rest after the long travel

What are the pressures faced by a cell after it leaves circulation and enters a foreign tissue? The new microenvironment is thought to ‘stress’ DTCs, although whether all tissues induce equal stress is unclear. However, cancers of different types exhibit discrete patterns of metastatic colonization, driven by specific gene expression and chemokine secretion patterns (Bos *et al*., 2009; Kang *et al*., 2003; Minn *et al*., 2005; Tabariès *et al*., 2015). This is commonly referred to as ‘organo‐tropism’ or cancer cell ‘homing’ (Nguyen *et al*., 2009; Wan *et al*., 2013). DTCs face an unfamiliar milieu of growth factors, ECM, and metabolic stresses upon extravasating (Aguirre‐Ghiso, 2007; Sosa *et al*., 2014) regardless of the destination tissue. An exception is when the primary tumor induces tissue remodeling from afar to create a premetastatic niche (Hoshino *et al*., 2015; Kaplan *et al*., 2005; Peinado *et al*., 2012; Psaila and Lyden, 2009). In this case, arrival within a familiar wound‐like milieu accelerates the dynamics of metastasis. But in healthy tissues, a potential result of these pressures is that DTCs are steered into a state of dormancy, a phrase coined by Willis in the context of tumor progression as early as 1934 (Willis, 1934). Indeed, perhaps most DTCs go through a phase of dormancy of some length as part of their normal biology (Aguirre‐Ghiso, 2007; Sosa *et al*., 2014).

Dormancy can imply one of two states that reflect two very different underlying biologies. Dormancy can be induced in tumor cells at the cellular level, where they exit the cell cycle and undergo a G0–G1 arrest or differentiation as a means to avoid replication in the absence of appropriate growth factor and adhesion signaling (Aguirre‐Ghiso, 2007; Ghajar, 2015) (Fig. 1, Step 6a). However, dormancy can also be induced at the population level due to the presence of cell‐depleting phenomena (such as apoptosis induced by diffusion limitations and/or immunosurveillance) that counter the proliferation of DTC masses (Aguirre‐Ghiso, 2007; Folkman, 1971; Gimbrone *et al*., 1972; Rakhra *et al*., 2010) (Fig. 1, Step 6b). Theoretically, these two dormancy types are not independent of one another and may indeed reflect two separate barriers that a DTC must overcome in order to progress toward a full metastatic lesion.

### 6.2 DTCs and their time of arrival at distant sites

Linear progression requires that DTCs be derived from the primary tumor at or around the time of detection (Klein, 2009; Klein *et al*., 2008). Due to the popularity of the ‘Vogelgram (Fearon and Vogelstein, 1990)’, many assume this to be the case. If so, DTCs should contain most/all of the mutations present in the primary tumor at the time of detection, and accumulate additional mutations over time.

Several lines of evidence are at odds with this paradigm, none more so than the presence of DTCs in the bone marrow of breast cancer patients with so‐called *in situ* disease (Gruber *et al*., 2016; Hüsemann *et al*., 2008; Sänger *et al*., 2011), and the emergence of metastases in both early‐stage cancers (T1M1 or T2M1) and in patients with cancer of unknown primary (Abbruzzese *et al*., 1994; Engel *et al*., 2003; Klein, 2009; van de Wouw *et al*., 2002). Thus, temporally speaking, early DTCs are exposed to selection pressures different from those experienced by the primary tumor, which influences their biology in a unique manner.

Several studies comparing DTC genotypes with that of primary tumor show that early DTCs are actually less evolved than the primary tumor. For example, in the case of breast cancer, bone marrow DTCs display fewer genetic abnormalities (chromosomal aberrations, subchromosomal allelic losses, and gene amplification) than cells from primary tumors as assayed by CGH; in fact, at this resolution, only half of bone marrow DTCs display abnormal karyograms, as opposed to 100% of cells from the primary tumor (Schardt *et al*., 2005; Schmidt‐Kittler *et al*., 2003). In the case of esophageal cancers, comparison of chromosomal rearrangements revealed significant heterogeneity between primary tumor cells and DTCs, and even between DTCs from different sites. These results argue for parallel evolution under potentially site‐specific selection pressures (Stoecklein *et al*., 2008).

The implications of parallel evolution are twofold: First, the therapeutic based on properties of the primary tumor (e.g., targeting certain receptors that have genetic amplifications) might be effective only against a subset DTCs. And second, DTCs occupy microenvironments totally distinct from that found in the primary tumor. The role that these ‘foreign’ microenvironments play in the biology of DTCs is thus a necessary consideration.

### 6.3 DTCs: a product of their (micro)environment

**Figure 2:**
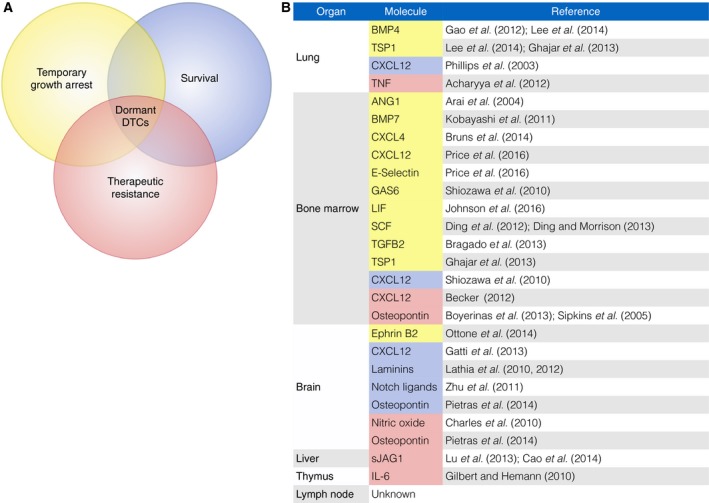
Characterization of dormant disseminated tumor cells. (A) Dormant tumor cells have three primary characteristics that distinguish them from other tumor cells and contribute to their metastasis‐initiating potential: the ability to survive in foreign and often hostile environments for a decade or more, temporary and reversible growth arrest, and resistance to therapies. (B) Dormant DTC characteristics are driven by tissue‐specific microenvironmental effectors. Molecules demonstrated to contribute to the DTC dormancy program, a significant fraction of which are derived from microvascular endothelium, are presented here by tissue type and by the dormant property endowed. Color coding matches that of the Venn diagram in panel A.

There are three characteristic properties of dormant DTCs – prolonged survival in a foreign microenvironment, reversible growth arrest at these sites, and resistance to targeted and cytotoxic treatment (Ghajar, 2015) (Fig. 2). DTCs from carcinomas originate from epithelia, and nearly all epithelial cells depend on adhesion to basement membrane in order to survive (Boudreau *et al*., 1995). As DTCs leave the primary tissue through either a hematogenous or a lymphatic route, the first basement membrane they come across at a distant site laminates the vascular endothelium (Fig. 1, Steps 4–5). Therefore, it is not surprising that dormant DTCs are found in close association with vascular basement membrane in various experimental models of breast cancer, lung cancer, and melanoma (Chambers *et al*., 2002; Ghajar *et al*., 2013; Kienast *et al*., 2010; Price *et al*., 2016) (Fig. 1, Step 6). For instance, in brain, fate mapping individual lung and melanoma DTCs via intravital microscopy revealed that adhesion to the abluminal surface of vasculature was a prerequisite for survival and ultimately progression to metastasis (Kienast *et al*., 2010). This microenvironment, called the perivascular niche (PVN), plays a dynamic role in normal tissue development and differentiation (Butler *et al*., 2010; Rafii *et al*., 2016). Stem cells from several different organs localize to the PVN, where they are growth‐regulated and maintained in a stem‐like state by endothelial‐derived/angiocrine factors (Butler *et al*., 2010; Christov *et al*., 2007; Goldman and Chen, 2011; Kiel *et al*., 2005; Xiao *et al*., 2013). Clear parallels can be drawn between stem cell maintenance and induction and maintenance of DTC quiescence by this niche.

While there is currently no conclusive evidence that the niche occupied by DTCs and stem cells is one and the same, there is evidence that these niches overlap (Ghajar, 2015). First, the size of the primary tumor correlates with the number of detectable CTCs, but not DTCs in bone marrow, suggesting that niches available for engraftment are limited at least in bone marrow (Braun *et al*., 2005; Klein, 2013; Zhang *et al*., 2003). Mouse model experiments demonstrate that human prostate cancer cells compete with hematopoietic stem cells (HSCs) for HSC niche occupancy and that dissemination can be compromised by reducing the niche size (Shiozawa *et al*., 2011). Once prostate DTCs occupy a HSC niche, they take on a cancer stem cell‐like phenotype (Shiozawa *et al*., 2016). This is in line with evidence that stem cell niches can induce so‐called terminally differentiated daughter cells to become stem‐like (Calabrese *et al*., 2007; Rompolas *et al*., 2013; Scadden, 2014; Tata *et al*., 2013). Second, mobilization of HSCs from bone marrow by the application of G‐CSF also leads to the eviction of DTCs (and a concordant spike in CTC number) in breast cancer and multiple myeloma patients, suggesting that there is substantial overlap in the physical, physiological, and biological control of these cells (Fischer *et al*., 2013). Finally, recent evidence suggests that DTCs modify stem cell niches in distant tissues, indicating that the properties of DTCs are, unsurprisingly, the result of dynamic interactions between DTCs and their niche. A detailed study of disseminated acute myeloid leukemia (AML) stem cells revealed that the arrival of AML stem cells in the bone marrow disrupts sympathetic nerves, destroying periarteriolar nestin^+^ HSC niche cells and ultimately depleting HSC niches (Hanoun *et al*., 2014). Interestingly, the leukemic bone marrow also shows an expansion of endothelial cells via proliferation (Hanoun *et al*., 2014), suggesting that aberrant perivascular AML stem cell niches might also expand to allow a greater number of DTCs to engraft, although this has not been tested.

Several known and hypothesized factors that confer DTCs with key properties have been discussed elsewhere, and it is interesting to note that the dormancy‐associated effect of many of these molecules is tissue specific (Ghajar, 2015) (Fig. 2). This likely speaks to the tissue specificity of the PVN. But one component common to every PVN is microvascular endothelium, and in the case of breast cancer, growth‐arrested DTCs are intimately associated with brain, lung, and bone marrow vasculature (Ghajar *et al*., 2013). This phenomenon is observed in patients (Price *et al*., 2016) and has been re‐created in 3D culture models (Ghajar *et al*., 2013). Endothelial‐derived thrombospondin‐1 (TSP‐1) is a pivotal player in the induction of tumor dormancy in lung and bone marrow. Bone morphogenetic protein (BMP)‐4 has been hypothesized to play a key role in inducing dormancy in the lung, although it is potentially relevant to note that autocrine BMP‐4 signaling results in TSP‐1 expression by lung endothelium (Gao *et al*., 2012; Lee *et al*., 2014). On the other hand, dormancy induction in bone marrow has been attributed to the action of stromal BMP‐7, transforming growth factor‐β2 (TGF‐β2), and leukemia inhibitory factor (LIF) in prostate cancer, head‐and‐neck squamous cell carcinoma, and breast cancer, respectively (Bragado *et al*., 2013; Johnson *et al*., 2016; Kobayashi *et al*., 2011). Similarly, factors that play a role in therapeutic resistance of DTCs in different niches may not completely overlap. Other known and hypothesized factors identified in different tissues are referenced in Fig. 2.

Eradicating the reservoir of minimal residual disease requires total elimination of DTC burden; thus, the final property of dormant DTCs – therapeutic resistance – is a particularly key hurdle to overcome. Many assume that cell cycle status is the reason that dormant DTCs resist chemotherapy, but there is currently no clear answer as to why DTCs are chemoresistant. A careful look at their biology and microenvironment provides some clues that perhaps cell cycle status is not the only answer.

The heterogeneity between DTCs and the primary tumor (a combination of early dissemination, site‐specific selection pressure, and parallel evolution) results in a substantial fraction of DTCs that do not express molecular targets derived from the primary tumor. For example, EGFR mutations did not correlate in more than 75% of paired primary tumors and metastases in a NSCLC cohort in a failed adjuvant clinical trial where EGFR was targeted using gefitinib (Goss *et al*., 2010; Gow *et al*., 2009). Additionally, pathways active in dormant DTCs such as the p38 pathway might supersede oncogenic pathways, making these cells resistant to targeted therapies (Adam *et al*., 2009; Polzer and Klein, 2013). Finally, the niche of dormant DTCs may also play a role in chemoresistance (Ghajar, 2015). Disseminated colorectal cancer cells expressing the putative cancer stem cell marker CD133 are enriched on the microvasculature of human liver and are sustained in the face of 5‐fluorouracil and oxaliplatin by cleaved Notch ligand jagged‐1 derived from liver endothelium (Lu *et al*., 2013). The soluble form of angiocrine Jagged‐1 in liver has also been implicated in doxorubicin resistance of lymphoma cells (Cao *et al*., 2014). The induction of certain cancer stem cell‐like properties may also underlie resistance mechanisms conferred by the PVN. The brain's PVN induces a stem cell‐like phenotype in proneural glioblastoma cells, resulting in expression of ABC transporter G family member 2 (ABCG2), known to actively efflux chemotherapeutics (Charles *et al*., 2010; Fletcher *et al*., 2010). Additionally, the DTC microenvironment itself may respond to chemotherapy and release factors that enable DTC survival (Acharyya *et al*., 2012; Sun *et al*., 2012). To understand the chemoresistance of these cells, efforts that take into account the timing of dissemination, the influence of the microenvironment on DTC survival, and response of the DTC microenvironment to treatment are needed.

## 7 Isolation and molecular and functional characterization of DTCs

Several tools, techniques, and platforms available for detection and characterization of DTCs have been reviewed exhaustively (e.g., Magbanua *et al*., 2015). In this section, we will provide a general overview of techniques and interesting findings from a subset of these studies.

### 7.1 DTC isolation

The molecular and functional characterization of DTCs is made difficult because their isolation invariably requires invasive procedures and positive selection. Detection of single DTCs in a noninvasive manner even with the use of high‐resolution imaging technologies is currently not possible in the clinic. Thus, current strategies rely on isolating DTCs from accessible tissue that lacks common epithelial tumor markers – primarily lymph node and bone marrow biopsy. Whole lymph node isolation and bone marrow biopsy samples are routinely performed in the clinic, and cytokeratins are currently used as standard markers for the detection of epithelial tumor cells in organs of mesenchymal origin. Additional markers such as proliferation‐associated Ki67 (Pantel *et al*., 1993), stem cell markers (CD44^high^ CD24^low^) (Balic *et al*., 2006), and HER2 (Schmidt‐Kittler *et al*., 2003) are also used to characterize DTCs. As new markers are identified and verified, they are increasingly being applied in multi‐marker fashion that is higher throughput than ICC/IHC alone. For example, using a system that detects transcripts of 38 genes (nCounter™ system), it was found that three patients with metastatic breast cancer had detectable levels of ERBB2 expression in their bone marrow despite their corresponding tumors being HER2/ERBB2 negative (Siddappa *et al*., 2013). This has clinical relevance as the Her2 gene is a common target for systemic therapy and these patients did not receive trastuzumab. Subsets of DTCs that express breast cancer stem cell markers (e.g., CD44^+^ CD24^−/low^) (Bartkowiak *et al*., 2010) have also been identified, although it is unclear whether this subset has greater metastasis‐initiating capacity than others.

### 7.2 Molecular characterization of DTCs

Single‐cell DNA sequencing technologies are increasingly being utilized to study DTCs. These allow the detection of small, novel, and copy‐neutral genetic changes. Comparing primary tumor cells and DTCs from two patients with breast cancer in this fashion revealed that although DTCs often carry the major chromosomal aberrations found in the primary tumor (whole arm gains/losses), they often carry unique genetic changes as well, further evidence of dynamic and divergent evolution (Møller *et al*., 2013). Whole transcriptome profiling using oligonucleotide microarray analysis revealed that the transcriptome signature of some prostate cancer bone marrow DTCs from patients with advanced disease clustered closely with that of DTCs from patients with no evidence of disease (Chéry *et al*., 2014), perhaps indicative of the bone marrow microenvironment's dominance over DTC gene expression. Efforts to correlate genetic and expression profiles in single DTCs (Gužvić *et al*., 2014) could potentially generate a specific expression signature to predict the metastasis‐initiating capacity of DTCs in different cancers.

### 7.3 Functional characterization of DTCs

Functional characterization (culture‐based and *in vivo* tumor/metastases‐forming assays) of DTCs is hampered mainly by the limited number of DTCs that can be isolated from patients. DTCs have been used to establish cell lines to address this problem. Although genetic changes may be initially maintained, the biology of DTC lines cannot be expected to fully recapitulate *in situ* DTC biology due to the lack of the full complement of microenvironmental factors. Nevertheless, important conclusions have been made from DTC lines. Analysis of DTCs from a number of different sites that either commonly support metastasis or are rarely affected by metastasis challenged the notion that tumor cells ‘home’ to specific tissues. Solitary MDA‐MB‐435 CL16 breast cancer cells isolated from metastasis‐free organs such as bone and spleen were shown to be just as tumorigenic and metastatic as cells isolated from preferred organs of metastasis such as lungs and lymph nodes (Suzuki *et al*., 2006), suggesting the need for a permissive microenvironment. DTC lines also provide further evidence of parallel progression. The proliferation rate of breast cancer cell lines established from M0 DTCs (i.e., patients without detectable metastatic disease) does not correlate with the proliferative potential of cells from the corresponding primary tumor (Gangnus *et al*., 2004). CGH analysis showed that the genomic profiles of these DTCs and their matched primary were very different, suggesting early dissemination and independent evolution of DTCs (Gangnus *et al*., 2004). Efforts are underway to test whether the dormancy phenotype can be established in cell lines by recapitulating the niche compartment that regulates DTC dormancy in each tissue. For breast cancer, this involves establishing microvascular niche cultures because dormant DTCs are often found in close association with endothelia – a phenomenon observed both in patients and in mouse models (Ghajar *et al*., 2013; Price *et al*., 2016). DTC biology, especially the influence of microenvironment on quiescence and re‐emergence, can thus be studied in tractable models that do not necessarily depend on establishment of cell lines derived from isolated DTCs.

## 8 Clinical relevance of DTCs: the persistent face of cancer

Disseminated tumor cells detected before and after adjuvant therapy, radiotherapy, or surgical resection of the primary tumor have been described as independent risk factors for relapse and death (Becker *et al*., 2007; Berg *et al*., 2007; Gruber *et al*., 2014; Hartkopf *et al*., 2013; Janni *et al*., 2011; Lilleby *et al*., 2013; Mathiesen *et al*., 2012; Morgan *et al*., 2009; Naume *et al*., 2014; Tjensvoll *et al*., 2012). Yet, despite DTCs predicting adverse outcomes, the majority of DTC‐positive patients (specifically, 2/3 for breast cancer) do not develop metastases (Braun *et al*., 2005). This suggests that the ability to take up residence in bone marrow is not sufficient to initiate metastases.

Factors that may help explain this outcome include heterogeneity in the timeline of dissemination, influence of the microenvironment, and largely unknown effects of adjuvant therapy on DTC phenotype. There may be an as yet unknown trigger of DTC re‐emergence that occurs in the right space and time in patients that develop metastases that does not occur in others. In culture and in zebrafish, neovascular tips ‘awaken’ dormant breast cancer cells (Ghajar *et al*., 2013) by depositing many of the factors known to cause aggressive growth of many cancers in the premetastatic niche (Psaila and Lyden, 2009). These data suggest that physiological stresses that induce neoangiogenesis, such as systemic inflammation or surgery, could cause dormant cells to re‐enter the cell cycle. Along these lines, it was shown in a retrospective study that early breast cancer relapse incidents in the 9‐ to 18‐month period after surgery are reduced approximately fivefold in patients taking preoperative nonsteroidal anti‐inflammatory drugs (Retsky *et al*., 2013), suggesting that systemic inflammation is a indeed a trigger. Additionally, there are a number of case studies involving surgery conducted on breast cancer survivors years after treatment that caused noncanonical metastatic outgrowth at the surgical site, such as in the vicinity of dental implants and the site of tracheotomy (Dib *et al*., 2007; Rotolo *et al*., 2013).

Overall, very little progress has been made on therapeutic strategies to address DTCs. The heterogeneity between the primary tumor and DTCs, and between DTCs at different sites, are doubtlessly contributing factors. Additionally, it has never been proven that DTCs are the source of future metastasis/metastases (Goss and Chambers, 2010; Meng *et al*., 2004; Uhr and Pantel, 2011). Ironically, the only proof is an effective therapy. Here, it is important to note that small trials targeting DTCs post‐treatment have been successful. Second‐line therapies such as bisphosphonates (Banys *et al*., 2013; Kasimir‐Bauer *et al*., 2016) or docetaxel (Naume *et al*., 2014) have led to increased survival for patients with breast cancer. The latter trial was particularly promising as the risk of recurrence for docetaxel‐treated patients effectively treated to eliminate DTCs was reduced to the level of patients who were initially DTC negative (Naume *et al*., 2014). These clinical successes in targeting DTCs are promising, but leave room to identify more specific strategies to eliminate DTCs with first‐line therapy. Another unexplored approach to disease management could involve prolonged induction of dormancy to make DTCs, dormant or otherwise, incapable of progressing from a growth‐arrested state (Ghajar, 2015).

Thus, deeper understanding of the factors that induce and sustain dormancy may open avenues to a) inhibit factors that trigger activation of dormant DTCs or b) sustain quiescence indefinitely (Ghajar, 2015). Analysis of the dormant niche via mass spectrometry, metabolomics, etc., may identify proteins and metabolites that induce dormancy and/or activation. Development of tissue‐specific niche models that recapitulate the cellular and molecular composition of native DTC niches are pivotal to better understand dynamic changes in niche composition under different pressures such as chemotherapy. Such efforts may reveal novel factors or signaling pathways involved in conferring and/or sustaining dormant properties. In this endeavor, the ideal therapeutic would be a prophylactic therapy administered as soon as the primary tumor is detected in order to keep the DTCs dormant. This strategy has been elaborated on elsewhere along with a careful consideration of clinical trial design (Ghajar, 2015).

## 9 Concluding remarks

In this review, we examined our current understanding of CTC and DTC biology and profiled technologies available to detect and molecularly characterize these cells. Additionally, we discussed the clinical relevance of CTCs and DTCs and posed several important questions and considerations – from technical caveats and challenges in analyzing DTCs and CTCs, to the need to address the current discrepancy in the timing of dissemination as a clinically relevant factor. Regardless, studying these cells through the use of more refined detection, isolation, and characterization technologies, and carefully designed animal models, has the potential to make CTCs and DTCs important diagnostic and prognostic biomarkers in disease management. Ultimately, however, our aim should be to either eliminate or permanently incapacitate these cells so that they are no longer able to progress toward lethal metastases. So how do we progress from a lack of therapies that specifically target CTC and DTCs to making metastasis prevention by targeting the relevant population(s) a reality?

As is evident from Fig. 2, we still have much to learn about the dormant niche. What sustains the survival of DTCs from different cancers within different tissues, how do tissue microenvironments steer DTCs into a quiescent state, and how do these controls go awry to foster DTC re‐emergence? The hope is to identify universal mechanisms that will allow us to target DTCs throughout the body, but in the absence of a ubiquitous control mechanism, we will need tissue‐specific approaches that can be applied to patients with cancers prone to metastasize to restricted sites (e.g., pancreatic cancer to the liver) or cancer subtypes with a defined risk of metastasis to a particular site (e.g., ER‐positive breast cancer to the bone).

Once we realize the above, we still have to answer the most relevant question: Whom do we treat? In patients with breast cancer, it is well established that DTC‐positive bone marrow aspirates reflect a greatly enhanced risk of metastasis. But still, only one‐third of this population will progress, meaning that applying DTC‐targeted therapies to all marrow‐positive breast cancer patients would result in overtreatment. This is not acceptable. Profiling DTCs or CTCs derived from DTCs at the molecular level could reveal indicators of metastasis‐initiating potential, allowing us to stratify DTC‐positive patients further. Then, the goal would be to treat only this subset of patients and eliminate metastasis‐initiating cells so that they perish of causes unrelated to cancer.

Ultimately, it is important to remember the old adage, ‘an ounce of prevention is worth a pound of cure.’ Applying this to CTCs and DTCs to prevent metastasis has the potential to prolong the lives of many cancer survivors. The time is ripe to commit to this approach.
